# Characterization and functional properties of conjugates of rice protein with exopolysaccharides from *Arthrobacter* ps‐5 by Maillard reaction

**DOI:** 10.1002/fsn3.2336

**Published:** 2021-07-11

**Authors:** Yuguang Zhao, Shuhong Ye, Huiping Wan, Xingxing Zhang, Mingqi Sun

**Affiliations:** ^1^ School of Food Science and Technology Dalian Polytechnic University Dalian China; ^2^ School of Light Industry and Chemistry Engineering Dalian Polytechnic University Dalian China

**Keywords:** conjugates, exopolysaccharide, functional properties, Maillard reaction, rice protein

## Abstract

The study examined the potential nutritive value of rice protein (RP) through Maillard reaction. Structures and properties of synthetic conjugates of RP and exopolysaccharide (EPS) from *Arthrobacter* ps‐5 were investigated systematically. Fluorescence characteristics and high molecular weight compounds appeared in Maillard reaction products (MRPs). Moreover, EPS or its degradation products in the form of covalent bond cross‐linked with RP were identified, where –NH_2_ disappeared and C=O, C=N and C–N increased. Determination of free –SH residues suggested mutual conversion between disulfide bonds and sulfhydryl groups occurred during Maillard reaction. HPLC analysis identified conjugates with different molecular weight, where melanoprotein was formed by covalent bonds. As RP conjugated with EPS, the molecules spread out and changed the spatial structure. Functional properties of MRPs, including solubility, foaming activity, emulsifying ability and resistance to oxidation, were greatly improved. The study has discovered an efficient method for increasing the application value of plant protein.

## Graphical abstract

The rice protein and exopolysaccharide were grafted by Maillard reaction and the structural changes and functional properties of the Maillard reaction products were studied. Functional properties of MRPs, including solubility, foaming activity, emulsifying ability, and resistance to oxidation, were greatly improved. The study provided an efficient method for increasing the application value of plant protein.

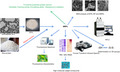

## 1 INTRODUCTION

As the main staple diet for more than half of the world's population, rice is one of the most important food crops all over the world and a reliable source of protein. Protein fraction from the rice dregs and defatted rice bran residues, however, are usually discarded (Piotrowicz & Salas‐mellado, 2017). Rice protein (RP) has gained much attention recently because of its high yield, high nutritional value and economic value (Zhang et al., 2020). However, the poor solubility of rice protein limits its further application (Wang et al., 2020). Over 80% of rice proteins are glutelins with low solubility at neutral pH. As a result the presence of other functional properties, such as foaming, gelling and emulsifying, is limited (Agboola & Mills, 2005).

Maillard reaction is named by the French chemist Louis Maillard. It involves not only a reaction pathway, but also various complex reaction networks of carbonyl and amino compounds, such as reducing sugars and amino acids, amino groups of peptides, or proteins. The Maillard reaction, also called nonenzymatic browning reaction, plays an important role in food chemistry and tobacco industry. The advanced compounds of sugar–protein reactions are referred to as advanced glycation end products (AGEs) and melanoidins (or melanoproteins) (Chellan et al., 1999). The Maillard reaction is a nonenzymatic browning reaction occurring between amino and carbonyl groups during heat treatment or cooking and is regarded as one of the most important reactions during food processing and storage (Cui et al., 2017).

Generally, the Maillard reaction can be divided into three stages (Naresh et al., 2017). In the initial stage, an amino of amino acids or proteins condenses with a carbonyl of reducing sugars and obtains the initial MRPs‐Amadori compounds followed by the Amadori rearrangement. Subsequently, a range of reactions takes place, including enolization and Strecker degradation. In this step, Amadori compounds decompose to numerous flavors and aromas at high temperatures. The final stage involves the formation of brown nitrogenous polymers named melanoidins. The complexity of the reaction leads to the diversity of MRPs, while most MRPs have good broad pharmacological applications.

As the demand for relatively inexpensive sources of proteins that can be incorporated in value‐added food products is increasing, many recent research studies involve various sources of plant proteins (Gorinstein et al., 2002) that may help increase the nutritional value of food products at low cost. Milled rice is the major byproduct generated during milling and is further extracted for oil or used as manufactured feeds. The average content of protein in Milled rice is about 8%–13% (Nakase et al., 1996) and the protein is considered as one of the highest nutritive value among cereal proteins as for its colorlessness, bland taste and richness in essential amino acids, and hypoallergenic as well as hypocholesterolemic (Chrastil, 1992), suggesting that rice protein should be considered when the potential for rice's added value is investigated. This is especially the case when the low‐commercial value of some rice products, such as broken, chalky and debris rice or byproduct of rice starch, is considered. The values of these products could be significantly improved if the extracted rice protein's functionality is suitable for food manufacture.

In our previous studies, exopolysaccharides‐producing strain *Arthrobacter* ps‐5 from rhizosphere soil of Gingko biloba trees was obtained (Ye et al., 2014). The objective of the study was to prepare conjugates of rice protein with EPSs from *Arthrobacter* ps‐5, to improve functional properties of the Maillard reaction products to help increasing the application value of plant protein at low cost in practical applications.

## 2 MATERIAL AND METHODS

### 2.1 Materials and reagents

Milled rice grains were reclaimed from the local food processing factory. Other chemicals used were all analytical grades and purchased from Tianjin Kermel Chemical Reagent Company.

### 2.2 Preparation of rice protein

Rice protein from milled rice grains was separated and dissolved with a method of alkali hydrolysis and acid precipitation. Rice was pulped after immersed in deionized water for 12 hr. The slurry was suspended in 0.1 M NaOH (the ratio of slurry to NaOH solution was 1:3, w/w) at 40°C. The suspension was continuously stirred for 2 hr and then centrifuged at 1198 *g* for 30 min. The supernatant was removed with its pH adjusted to 5.5 using 0.1 M HCl and was left undisturbed for a cold precipitation overnight (4°C) before being carefully siphoned off from the precipitate. The precipitate was washed twice with deionized water. Moreover, the pH was neutralized to 7.0 with 0.1 M NaOH before lyophilized. The protein content of the lyophilized substrate was determined by the Kjeldahl method (Zhang et al., 2018).

### 2.3 Preparation of *Arthrobacter* ps‐5 exopolysaccharides

*Arthrobacter* ps‐5 isolated from rhizosphere soil of Gingko biloba trees was stored in the Microbiological Culture Collection Center of Dalian Polytechnic University.

EPSs from *Arthrobacter* ps‐5 were obtained by submerged culture. The liquid culture medium for *Arthrobacter* ps‐5 was predetermined according to our previous studies, consisting of 2.5% sucrose, 2.5% beef extract, 0.5% NaCl, 0.1% K_2_HPO_4_, 0.1% MgSO_4_, 0.05% KH_2_PO_4_ and 0.05%MnSO_4_. *Arthrobacter* ps‐5 was propagated in 250 ml conical flasks with 50 ml of liquid medium and shaken at 160 rpm for 48 hr on a rotary shaker. The fermentation temperature, initial pH, inoculation proportion were 30°C, 7.5 and 4.0% (v/v) respectively.

The fermentation liquid of *Arthrobacter* ps‐5 was obtained from the cultivated broth using centrifugation at 1923 *g* for 20 min, and a threefold of the volume of 95% (v/v) ethanol was added, and then kept left at 4°C for overnight. The isolated crude EPS was dissolved distilled water and further treated with Sevag reagent (chloroform: n‐butanol at 5:1, v/v) for 3 times to remove the residual protein. The EPS in supernatant was purified again by ethanol and left overnight. The resulting precipitate was redissolved in distilled water and dialyzed (MWCO 7000) using running tab water for 48 hr and distilled water for another 48 hr (Liu et al., 2010). The dialyzed solution was concentrated and freeze‐dried, achieving 80% purity of EPS. The level of endotoxin in the EPS (0.115 EU/ml, 1.704 EU/ml of positive control) was tested, verifying that the EPS can be applied in food and medical industries.

### 2.4 Preparation and purification of Maillard conjugates

Rice protein was dispersed in 200 ml deionized water and later stirred at 50°C with the pH adjusted to 12 with 0.1 M NaOH. EPS was added to the dispersion with a ratio of 4.91:1 (the total solids content was 2.5% w/v) with the pH of dispersion readjusted to pH 12.04 with 0.1 M NaOH. The samples were heated in a water bath at 88.4°C for 1.5 hr, and then immediately placed in an ice‐bath to cool down. Finally MRPs were obtained by freeze‐dried and used for further experiments.

A certain amount of freeze‐dried samples of rice protein and its grafts were stirred to dissolved in Tris‐HCl buffer solution (containing 0.4 mol/L NaCl at a concentration of 5 mg/ml) at pH 9.5, centrifuged 2131 *g* 15 min and then taken for 10 ml column chromatography (Sepharose CL‐6B column, 50 cm ×2.5 cm, elution rate 6 ml/10 min). The Tris‐HCl buffer with an eluent of 0.1 mol/L was detected at 280 and 420 nm respectively (Laura et al., 2007). Solutions with absorbance values at both 280 and 420 nm were collected and dialyzed in distilled water and the resulting dialysate was freeze‐dried to make dry powder, which was the purified graft sample.

### 2.5 Determination of degree of glycation

Degree of glycation was determined based on a spectrophotometric assay using Orhto‐Phthalic Aldehyde (OPA) method (Xin et al., 2009). Solution CA was prepared by dissolving 40 mg OPA in 1.0 ml of methanol and 3.0 ml dd H_2_O, while Solution CB was prepared by mixing 25 ml of 100 mM sodium tetraborate, 2.5 ml of 20% sodium dodecyl sulfate (SDS) and 100 μl of mercaptoethanol and diluting with water to a final volume of 50 ml. 200 μl of sample solution was added to a mixture of 0.3 ml CA and 3.7 ml CB. The solution was mixed briefly and incubated for 2 min at ambient temperature, and the absorbance was read at 340 nm using a vis spectrophotometer (752N UV). The blank was determined where dd H_2_O was used instead of sample solution. A calibration curve was obtained by using 0.25 μl 2 mM L‐lysine as a standard and the degree of glycation was calculated using the following equation (Achouri et al., 2005).

(1) DG(%)=(Ac‐Aa)/Ac×100%

where Ac was absorbance of the control and Aa was absorbance of the sample.

### 2.6 Analysis of MRP by fluorescence spectrum

Fluorescence spectra of the MRP and rice protein were analyzed using a fluorescence spectrometer (Perkin Elmer LS‐55, USA) (Gorinstein et al., 2001; Morales & Boekel, 1997). The samples were dissolved in redistilled water and the following recordings were observed: the emission wavelength was at 420 nm; the excitation was scanned from 300 to 400 nm by fluorescence excitation spectra; the emission was scanned from 370 to 570 nm. Both excitation and emission slits were set to 5 nm under the temperature of 20°C and the scan rate of 120 nm/min.

### 2.7 Sodium dodecyl sulfate‐polyacrylamide gel electrophoresis (SDS‐PAGE)

Sodium dodecyl sulfate‐polyacrylamide gel electrophoresis was performed using 12% separating gel and 5% stacking gel with vertical slab polyacrylamide gel electrophoresis. Gels were stained with Coomassie Brilliant Blue R‐250 and Periodic Acids Schiff respectively. Destaining was conducted with a solution of methanol and acetic acid (Bayram et al., 2008).

### 2.8 Determination of Infrared spectrum (FTIR)

An aliquot of MRPs, rice protein and exopolysaccharides were added to potassium bromide (KBr) powder and pressed into laminate, respectively. Infrared spectra of the samples were obtained by an FTIR spectrometer (Perkin Elmer Spectrum One‐B, USA).

### 2.9 Determination of sulfhydryl groups

Sample Ⅰ (10 ml) consisted of 75 mg RP and 4.7 g guanidine hydrochloride and Sample Ⅱ (10 ml) consisted of 75 mg MRPs and 4.7 g guanidine hydrochloride. Measurement of sulfhydryl groups (–SH) was performed by using Ellman's reagent (Friedman, 1994). 4 ml mixture containing 0.1 mol/L Tris‐glycine buffer, 8 mol/L urea, 0.01 mol/L EDTA and 5 mol/L guanidine hydrochloride was added to 1 ml sample solution. After incubation at 40°C for 30 min, 50 μl of 5,5′‐dithiobis solution was added to reaction solution, which was then incubated at 25°C for 10 min. The absorbance was read at 412 nm. The total ‐SH residues were calculated as follows (Beveridge et al., 1974).

(2) $$‐SH\left(\mu mol/g\right)=\left(73.53\times A_{412}\times D\right)/C$$

where *A*_412_ was the absorbance at 412 nm. *D* was the dilution factor, 5.02. *C* was the concentration of samples, 7.5 mg/ml.

### 2.10 Estimation of molecular weight (MW) distribution

The molecular weight distributions of rice protein and MRPs were estimated by HPLC (Waters 2695 Alliance, Waters Inc., USA), Waters liquid Chromatography system equipped with a Ultrahydrogel™ 1,000 column (7.8 × 300 mm, Waters, USA). The column was maintained at 30°C and operated at a flow rate of 0.5 ml/min with 50 mM carbonate buffer (pH 9.5) and the effluent was monitored at 280 nm using a refractive index detector (2414 Waters Inc., USA). The samples were dissolved in the mobile phase to reach a final concentration of 0.1% (w/v protein) and filtrated with 0.45 μm filter membrane.

### 2.11 Scanning Electron Microscopy (SEM)

Maillard reaction products were laid on strips of double faced carbon tape, which were fixed to aluminum stubs with 1% of osmium tetroxide for 3–4 hr. Then the sample was sputtered coated with gold using Ion Sputter Coater for 30 s. The images were captured at 5 kV accelerating voltage by a model TM 3000 high vacuum SEM (JFC‐1600, LV, Japan).

### 2.12 Determination of functional properties of Maillard reaction products

#### 2.12.1 Determination of Solubility

Protein and MRPs dispersions (1%, w/v) were stirred in deionized water with magnetic stirring for 30 min, and then the pH was adjusted to 7 with 0.5 M HCl or 0.5 M NaOH. The pH was readjusted when necessary during the 30 min stirring process. Subsequently the dispersions were centrifuged at 10,000 *g* at 20°C for 20 min and the protein content was determined by Lowry's method using BSA as the Standard after appropriate dilution (AOAC, 1990). The solubility was expressed as grams of soluble proteins per 100 g proteins.

#### 2.12.2 Determination of foaming capacity and foaming stability

The sample (0.16 g) was dissolved in 20 ml Tris‐HCl buffer (various pH) and the consequent mixture was homogenized at 13320 *g* for 1 min (Karina et al., 2011). Formulae (Equation 3) and (Equation 4) were used in the calculation of foam ability (FA) and foaming stability (FS) of samples.

(3) $$FA=\frac{V_{0}}{20}\times 100\%$$

(4) $$FS=\frac{V_{10}}{V_{0}}\times 100\%$$

where V_0_ was the foam volume at the 0th min, and *V*_10_ was the foam volume at the 10th min after homogenization.

#### 2.12.3 Determination of emulsion ability and emulsion stability

The sample (0.1 g) was dissolved in 15 ml Tris‐HCl buffer solution. Five milliliter of soybean oil was added, and the system was dispersed at the speed of 10,000 rpm to prepare the emulsion (Ulrike et al., 2005). Emulsion stability was calculated based on the following formula.

(5) $$EA/\left(m^{2}/g\right)=\frac{2\times 2.303}{C\times \left(1‐\varphi \right)\times 10^{4}}\times A_{0}\times D$$

(6) $$ES=\frac{A_{10}}{A_{0}}\times 100\%$$

where absorbance value A_0_ measured at the 0th min was the emulsion ability (EA); A_10_ was the emulsion ability at the 10th min after the emulsification.

### 2.13 In vitro antioxidant activity

#### 2.13.1 Determination of reducing power

The reducing power of the Maillard reaction products (MRPs) was determined according to the method of Dorman (Dorman & Hiltunen, 2004) with modifications. 1 ml of MRPs was mixed with 2.5 ml of 0.2 M sodium phosphate buffer (pH 6.6) and 2.5 ml of 1% potassium ferricyanide (K_3_Fe(CN)_6_). The reaction mixtures were incubated in a temperature‐controlled water bath at 50°C for 20 min, followed by the addition of 2.5 ml of 10% trichloroacetic acid (TCA) after the mixtures were cooled to room temperature. The mixtures were then centrifuged at 4,000 rpm for 10 min while the supernatant obtained (2.5 ml) was treated with 2.5 ml of distilled water and 0.5 ml of 0.1% FeCl_3_. The absorbance of the reaction mixture was measured at 700 nm with 752N UV‐vis spectrophotometer. Results were the average of three measurements and expressed as absorbance units (AU).

#### 2.13.2 Determination of DPPH radical scavenging activity

The DPPH radical scavenging ability of MRPs was carried out according to the method of Ye (Ye et al.,. 2008) with slight modifications as a 0.2 mM of ethanolic DPPH·solution was prepared. The initial absorbance of the DPPH·in ethanol was measured at 517 nm and was not changed throughout the period of assay. An aliquot (2.0ml) of each sample was added to 2.0 ml of ethanolic DPPH·solution. Discolorations were measured at 517 nm after incubation for 30 min at room temperature in the dark and measurements were performed at least in triplicate. The percentage of DPPH·which was scavenged was calculated using the following formula:

(7) $$\text{Scavenging}\left(\%\right)=\left(1‐A_{\text{sample}}/A_{\text{control}}\right)\times 100\%$$

where *A*_sample_ was the absorbance in the presence of the sample, *A*_blank_ was the absorbance in the absence of the DPPH·solution, *A*_control_ was the absorbance in the absence of the sample.

#### 2.13.3 Determination of hydroxyl radical (OH) scavenging activity

The hydroxyl radical scavenging activity of samples of MRPs was measured using a modified method (Sun et al., 2011). In particular, hydroxyl radicals were generated in a solution of 0.1 M EDTANa_2_‐Fe^2+^ (1.0 ml), 6% H_2_O_2_ (0.8 ml), 0.1 ml safranine T (0.52 mg/ml) and 1.0 ml sodium phosphate buffer (150 mM, pH 7.4). Samples were incubated for 30 min at 40°C and hydroxyl radicals were detected by monitoring absorbance at 520 nm. The sample was substituted with distilled water and sodium phosphate buffer replaced H_2_O_2_. The capability of hydroxyl radical scavenging was calculated using the following equation:

(8) $$\text{Scavenging effect}\left(\%\right)=\left(1‐A_{\text{sample}}/A_{\text{control}}\right)\times 100\%$$

where *IC_50_* value (mg/ml) is the concentration at which the scavenging activity is 50%.

#### 2.13.4 Determination of superoxide anion (·O_2_^‐^) scavenging activity

Scavenging of superoxide radicals was assessed according to Yin Chen with some modification. In brief, 4.5 ml of 0.05 mol/L Tris–HCl buffer (pH 8.2) and 0.1 ml of sample solution at different concentrations were incubated at 25°C for 10 min, and 400 μl of pyrogallol at the same temperature was added to the mixture, and the reaction was allowed to proceed at 25°C for 4 min. The reaction was terminated by addition of 0.5 ml of HCl. The absorbance of the mixture was measured at 320 nm against the blank. Scavenging of superoxide radicals was calculated according to:

(9) $$\text{Scavenging ability(}\%)=(1‐A_{\text{sample}}/A_{\text{control}})\times 100\%$$

where *A*_control_ was the absorbance of control without the tested samples, *A*_sample_ was the absorbance in the presence of the tested samples, *IC_50_* value (mg/ml) was the concentration at which the scavenging activity was 50%.

#### 2.13.5 Statistical analysis

Measurements were expressed as mean values of at least three parallel replicates ±standard deviation. Data were subjected to an analysis of variance using SPSS statistical software. Difference was considered to be significant level at *p* < .05.

## 3 RESULTS AND DISCUSSION

### 3.1 Preparation of EPS‐RP MRPs

Rice protein–polysaccharide conjugates were prepared by hydrothermal Maillard reaction. The experimental degree of glycation at the optimum level was 46.8%.

**FIGURE 1:**
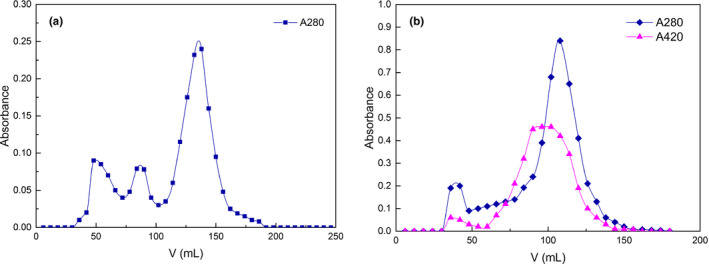
Chromatography of RP (a) and conjugates (b) in Sepharose CL‐6B

Conjugates were purified by Sepharose CL‐6B column chromatography (Figure 1a). At 280 nm there were three components in RP and two components in Maillard conjugates. The absence of the third component indicated that the components with small molecular weight decreased and the macromolecular components conjugated with EPS increased.

The collected solution of conjugates was further purified at 420 nm (Figure 1b). The main component (the second peak) was collected for further dialysis, freeze drying and structural analysis.

### 3.2 Analysis of fluorescence spectrum

The fluorescence was associated with the early stage of the Maillard reaction and the development of fluorescence compounds, which were considered to be precursors of brown pigments formed during Maillard reaction (Morales et al., 1997). The study (Kato et al., 1991) found that the typical spectral features of fluorescence material produced by Maillard reaction were excitation wavelength of 310–370 nm, emission wavelength of 400–440 nm. Since EPS and rice protein were the base of Maillard reaction, Fluorescence Spectrum was carried out and a maximum fluorescence response of MRPs was at excitation wavelength of 336 nm and emission wavelength of 420 nm, in accordance with fluorescence characteristics of the Maillard conjugates (Obayasgi et al., 1996), indicating that the conjugation of EPS and rice protein happened and the conjugates had fluorescence properties.

### 3.3 Analysis of sodium dodecyl sulfate‐polyacrylamide gel electrophoresis

Sodium dodecyl sulfate‐polyacrylamide gel electrophoresis is considered as one of reliable and feasible methods to identify covalent coupling between proteins and polysaccharides (Akhtar & Dickinson, 2007; Shu et al., 1996), and is used for analysis of subunit molecular weight, since hydrogen bond of intramolecular and intermolecular can be fractured by SDS, meanwhile secondary and tertiary structures of protein are destroyed and the original state of charge is lost.

**FIGURE 2:**
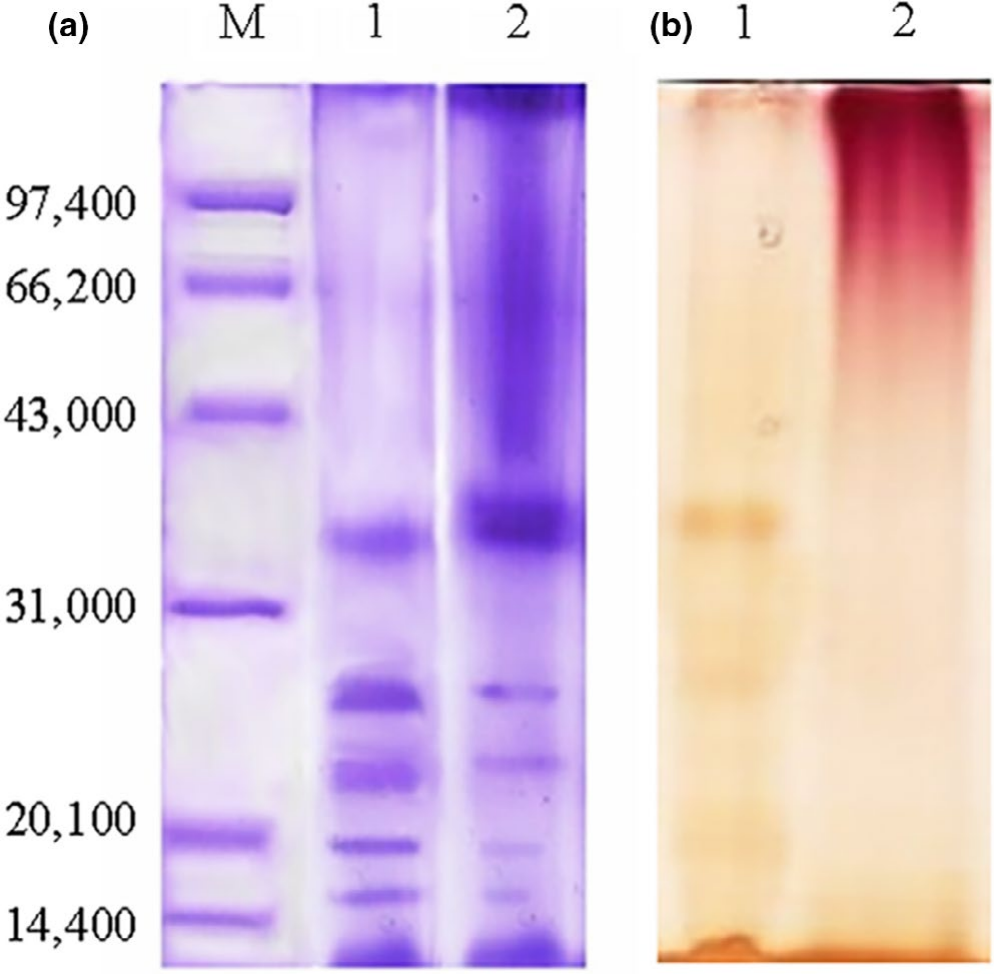
SDS‐PAGE of rice protein and MRP. "A: Coomassie blue stain. B: PAS stain "The labelled lanes are: (M) marker proteins; (1) rice protein; (2) MRP

Periodic Acid‐Schiff (PAS) reaction is an important method to identify the binding of saccharide in protein. In this study protein components were identified with a Coomassie blue stain and polysaccharide components with PAS stain (Zacharius et al., 1969). Bands intensity of smaller molecular weight decreased and some bands intensity of a high molecular weight increased (Figure 2), indicating that the Mallarid reaction mainly occurred in the subunits with small molecular weight. Therefore, results of PAS reaction showed that the band of a high molecular weight appeared near the stacking gel, which was potentially caused by the covalent binding of rice protein and exopolysaccharide and the increase of relative molecular weight. This observation further indicated that the RP‐RPS conjugate reaction led to the formation of high molecular weight compounds (Figure 2).

### 3.4 Analysis of fourier transform infrared spectrometer

Fourier transform infrared spectrometer analysis is a particularly useful technique for the identify bands attributable to functional groups present in protein–carbohydrate systems (Farhat et al., 1998), as there are several readily identifiable regions of the mid‐infrared spectrum where the chemical fingerprints of carbohydrates and proteins do not overlap significantly (Turner et al., 2002).

**FIGURE 3:**
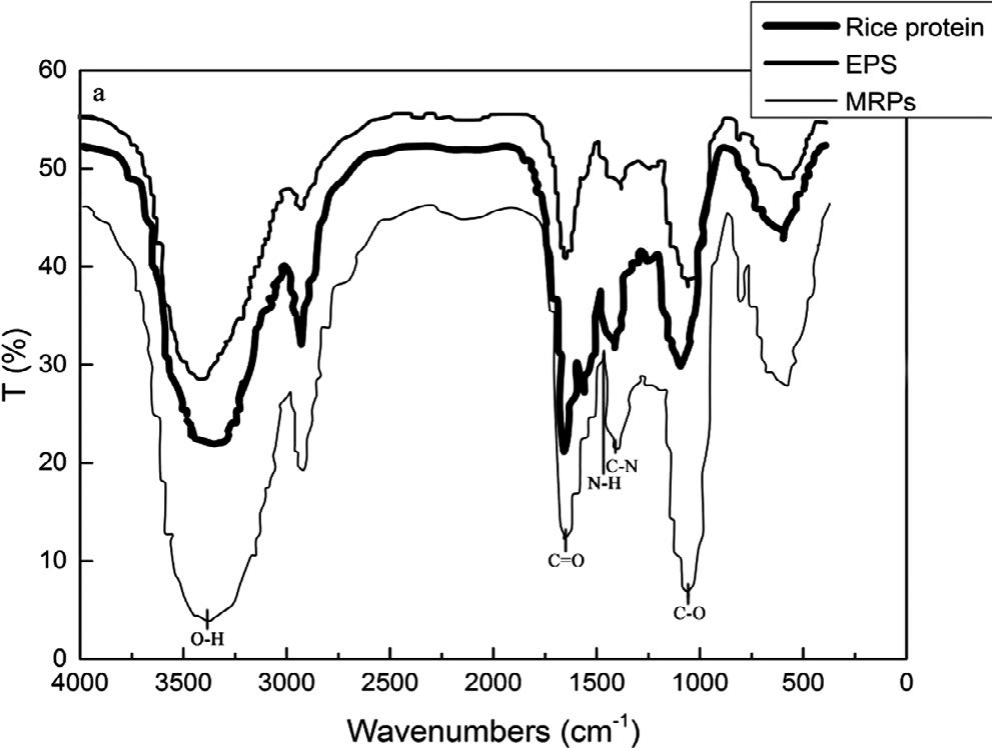
Infrared spectra of rice protein, EPS and MRP

These most distinctive spectral bands are in accordance with proteins that are the strong amide I and II bands at 1636–1680 cm^−1^ and 1533–1559 cm^−1^ respectively (Oliver et al., 2009). For carbohydrates, the characteristic absorption bands in the region of 1180–953 cm^−1^ are assigned to the stretching vibration of C–C and C–O and the bending mode of C–H bonds, which are usually referred as the “saccharide” absorption bands and are the most intense bands in the mid‐infrared spectrum (Cael & Koenig, 1974). These absorptions of most proteins are weak in the spectra (Thaís F et al., 2009) as can be seen in Figure 3, in which the absorptions of the region of 1180–953 cm^−1^ increased in MRP, indicating that there were polysaccharides attached to rice protein.

Polysaccharide bands are assigned to O–H stretching (3,291 cm^−1^), C–O stretching (1,034 cm^−1^), C–O–C stretching (1,179 cm^−1^) (Oliver et al., 2009). The intensity of the bands at 3,480–3,440 cm^−1^, 1,700–1,300 cm^−1^, 1,260–1,000 cm^−1^ gradually increased when EPS was added to rice protein. These changes reflected the crosslinking reaction between EPS and rice protein and the chemical changes in rice protein during the Maillard reaction would lead to several changes in the mid‐IR spectrum as a result of the consumption of some functional groups and the appearance of others. Functional groups including NH_2_ (N‐H, absorption peaks at 1,640–1,560 cm^−1^) were lost, especially those from lysine (Farhat et al., 1998), while the amount of those associated with MRPs, such as the Amadori compound (C=O, absorption peaks at 1,750–1,700 cm^−1^), Schiff base (C=N, absorption peaks at 1,690–1,640 cm^−1^), and pyrazines (C–N, absorption signals at 1,420–1,400 cm^−1^), would be increased by conjugation reaction. It can be concluded that carbohydrate or its degradation products in the form of covalent bond cross‐linked with rice protein.

### 3.5 Determination of –SH groups

Disulfide bond is very important for proteins to keep the molecular structure and is related to the biological activity of protein higher structure. Especially, –SH and ‐S‐S‐ groups in proteins play an important role in improving food functional properties (Friedman, 1994).

Based on Section 2.10, the contents of free ‐SH groups in rice protein and EPS were 3.494 μmol/g and 22.4 μmol/g, respectively. The results suggested that the content of free ‐SH residues was increased and mutual conversion between disulfide bonds and sulfhydryl groups occurred during the Maillard reaction, possibly due to the reducibility of EPS and the fracture of the ‐S‐S‐ groups of rice protein under the heating and reducing conditions. Because of the reduction of ‐S‐S‐ groups, the spatial structure of proteins became loose and the stability of proteins was decreased. Due to the reducibility of EPS, disulfide bonds in rice protein would break under the conditions of heating and reduction, leading to the decrease in the content of disulfide bond, the destruction of the spatial structure of rice protein and the reduction of stability (Gekko et al., 2003)..

### 3.6 Molecular weight distribution of MRPs

**FIGURE 4:**
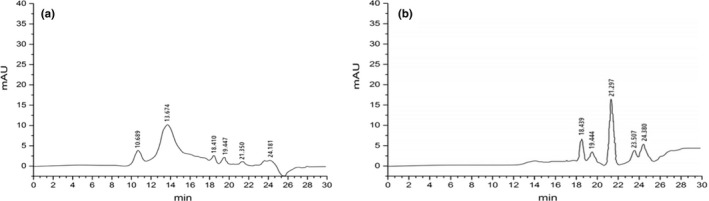
Distribution of molecular weight of rice protein (a) and MRP (b) by HPLC

Maillard conjugates are a series of products with different molecular weight distribution. As shown in Figure 4a and b, new peaks appeared in correspondence to small molecules. With the decrease of small molecule compounds, the number of high molecular weight compounds increased. Meanwhile, caramelization of EPS was conducted through heating EPS at a certain pH in case its degradation products induce the formation of low and medium molecular weight compounds. Macromolecular compounds (melanoprotein) were produced by covalent binding of rice protein with polysaccharides or degradation products of polysaccharides. According to the results of SDS‐PAGE, production of macromolecular compounds was not caused by aggregation of molecules, but by the formation of stable covalent bonds.

### 3.7 SEM analysis

**FIGURE 5:**
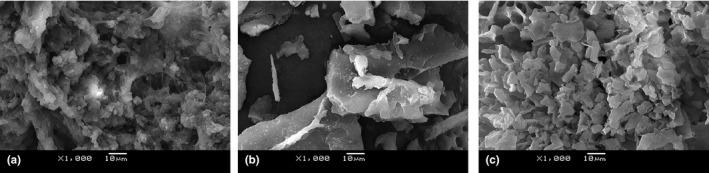
SEM analysis of EPS (a), RP (b) and MRPs (c)

The ultrastructure of proteins reflects the aggregation state of protein molecules and plays an important role in the functional properties of proteins. In this study, the aggregation of EPS, RP and MRPs was observed by SEM. As shown in Figure 5, the Maillard aggregates dispersed after conjugating modification, and formed even tiny particles, possibly due to the steric block effect of polysaccharides. After covalent binding of RP and EPS, the molecules spread out, reducing the molecular aggregation and changing the spatial structure of rice protein, which can be used to improve the texture of food.

### 3.8 Analysis of functional properties

#### 3.8.1 Solubility of MRPs in different pH

The solubility of protein plays a key role in its functional properties and requires consideration of the changes of solubility of the protein modified by EPS. The content of glutelin in RP is relatively high, while the disulfide bond in glutelin makes the polypeptide chain of the protein gather to form a tight molecule with a large number of hydrophobic groups inside. This structure results in the low solubility of RP, which further leads to the poor functional properties such as emulsion and foaming, and limits the application of RP in food industry. The changes of solubility of RP after modification were thus investigated.

**FIGURE 6:**
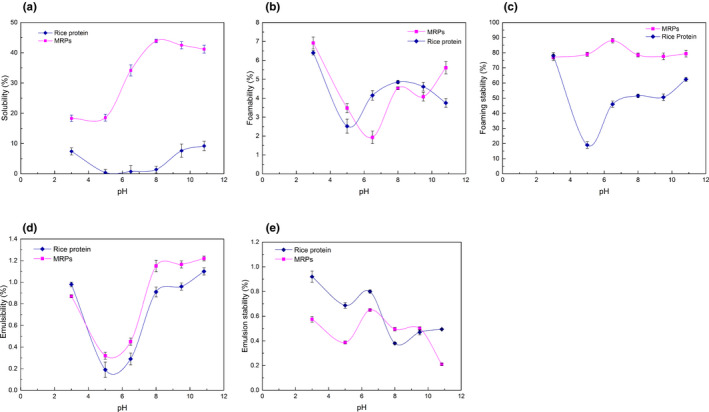
Physical properties of RP and MRPs (a Solubility of RP and MRPs)

As illustrated in Figure 6 a, near its isoelectric point pH 5.5, almost all of the conjugates were precipitated in solution, whereas the solubility far away from the isoelectric point increased slightly. After modification by Maillard reaction, the solubility of conjugates was greatly improved in different pH. At pH 3.0–5.0, the solubility of the complex was relatively low (about 20%) and then increased significantly. At pH 8.0, the solubility of rice protein was 1.41% and the solubility of graft reached a maximum of 43.91% and then decreased slightly.

In general, the solubility of rice protein‐EPS conjugates by Maillard reaction was greatly improved, from 3 times to nearly 10 times above the isoelectric point of RP, and 40 times above the pH 8.0. As a conclusion, conjugation with hydrophilic polysaccharides could improve the affinity between proteins and water molecules and restrict the interaction of proteins under unfavorable conditions (Jiménez‐Castaño et al., 2005). Due to the Maillard reaction, RP and EPS or its degradation products covalently combined, and with the introduction of hydroxyl, RP molecules hydrophilic groups increased, enhancing hydrophilicity, and increased solubility.

#### 3.8.2 Foaming properties of conjugates in different pH

The foaming property of protein is a significant functional property applied to food processing. Many processed foods, such as whipped cream, cake, bread, ice cream, etc., are foam products.

It can be seen from Figure 6 b that the foaming properties of the conjugates had not been significantly improved. When the pH was below 5.5 and over 10.0, the foaming properties of RP were higher than the properties of conjugates, especially when the pH is 6.5, where its foaming property is doubled. This pH range was suitable for the above foamed foods. Therefore, the application of conjugates in these foamed foods was better than RP.

As can be seen from Figure 6c, the foaming stability of conjugates was generally better than that of RP. Therefore, MRPs can be better applied to frothy food.

#### 3.8.3 Emulsification of MRPs in different pH

Protein products with high emulsifying ability can be widely used in beverage, dairy, chocolate, ice cream and other emulsifying products. Changes of emulsification of RP before and after Maillard modification were studied in order to develop natural emulsifier with high quality and low price. Emulsifying properties of proteins are related to the solubility, surface hydrophobicity and surface charge distribution of proteins. As stated in Figure 6d, the emulsion ability of conjugates at pH 4.0–10.83 was slightly higher than that of RP. The emulsifying activity of both decreased at initial stage, then increased, and finally reached the lowest at pH 5.0–6.0. When protein is used as an emulsifier, it is adsorbed on the surface of dispersed particles and its hydrophilic chain is extended into the water phase, which forms not only the adsorption film on the surface of particles to reduce their surface tension, but also a space protective layer around the particles to prevent the aggregation of particles. However, polysaccharides as stabilizers are mostly retained in the dispersing medium, and the rheology of the medium is changed by hydration or association to prevent the aggregation of colloidal particles. The covalently bonded protein‐polysaccharide grafts not only retain the surface activity of protein but also have the hydrophilic property of polysaccharide, hence can be used as natural emulsifiers and stabilizers (Kimio & Hiroki, 2018).

According to Figure 6, the hydrophilicity, solubility and emulsification of MRPs were increased. At the same time, the conformation change was an important way to improve the surface activity of emulsification. After the conjugation modification, the original tight structure in RP was damaged, and the hydrophobic groups hidden in the molecules were exposed. The lipophilicity was enhanced, and the number of charges also was increased. In addition, the oil droplets from approaching each other were prevented. All these observations indicated that the conjugates were easy to disperse on the oil–water interface.

As stated in Figure 6e, the emulsification stability of conjugates was higher than that of rice protein only at pH 7.0–9.5, and lower than that of rice protein in other pH ranges. At different pH, the addition of polysaccharide affected the emulsion stability by changing the viscosity of water phase in oil–water emulsion system.

#### 3.8.4 Antioxidant activities of MRPs

##### Reducing power

Reducing power of MRPs formed due to the Maillard reaction is shown in Table 1 It was seen that the original RP solution had weaker reducing power, whereas, after reaction, the reducing capacity of solution increased significantly. Reducing power of MRPs increased with the concentration of MRPs increased. In our initial studies, the reducing power of the EPS had been investigated. The reducing capacity of the EPS increased with increases in its concentration. The perfect linear correlation between the EPS concentration and its reducing capacity suggested a dose and effect relationship. When the concentration of EPS reached to 2.5 mg/ml, the corresponding absorbance at 700 nm was 0.679. The reducing capacity of MRPs is generally higher than original RP but to a great extent lower than EPS.

**TABLE 1:** Antioxidant status

Concentration (mg/ml)		Reducing power OD 700 nm	DPPH radical scavenging activity (%)	IC_50_	Concentration (mg/ml)	OH Radical scavenging activity (%)	IC_50_	Concentration (mg/ml)	O_2_^−^ Radical scavenging activity	IC_50_	
	1.0	0.171	2.905		2.0	21.2		42.0	26.0		
	2.0	0.246	14.25		4.0	31.0		44.0	31.6		
	4.0	0.383	24.52	7.536	6.0	37.5	9.411	46.0	37.1	49.989	
	6.0	0.556	31.11		8.0	44.0		48.0	43.8		
	8.0	0.686	35.095		10.0	52.2		50.0	51.8		
MRPs	1.0	0.270	16.97		1.0	34.6		8.0	28.8		
	2.0	0.382	42.53		2.0	43.9		10.0	36.8		
	4.0	0.586	53.74	3.340	3.0	52.6	2.547	12.0	47.1	12.488	
	6.0	0.700	61.435		4.0	66.5		14.0	55.0		
	8.0	0.808	68.625		5.0	76.7		16.0	69.5		
EPS	0.5	25.9	7.13		0.015	25.9		0.05	22.8		
	1.0	30.5	9.37		0.030	30.5		0.10	39.4		
	1.5	37.9	22.12	0.330	0.060	37.9	0.161	0.15	59.5	0.128	
	2.0	44.6	28.33		0.120	44.6		0.20	77.4		
	2.5	63.1	35.83		0.240	63.1		0.25	88.0		

##### DPPH radical scavenging activity (DPPH)

The relatively stable DPPH radical has been widely used to test the ability of compounds to act as free radical scavengers or hydrogen donors and thus to evaluate the antioxidant activity (Jao & Ko, 2002). The DPPH radical was scavenged by MRPs by donation of hydrogen to form a stable DPPH‐H molecule (Matthaus, 2002). The color changed from purple to yellow by acceptance of a hydrogen atom from MRPs. The results of DPPH radical scavenging activities are shown in Table 1. According to the results summarized in Table 1, the initial DPPH scavenging activity of RP solution was approximately 35.1% when the RP concentration reached to 8 mg/ml, However, at the low concentration of 2 mg/ml, the scavenging effect of MRPs was 42.5%. When the concentration of EPS reached to 0.3 mg/ml, the inhibitory of DPPH radical is 36.5%.

##### Hydroxyl radical scavenging activity

The hydroxyl radical is the most reactive of species and induces most severe damage to adjacent biomolecules, resulting in lipid peroxidation in biological systems. In the present study the Fenton reaction system was used in deoxyribose degradation by generating hydroxyl radicals. The treatment of deoxyribose with Fenton reaction reagent resulted in a high rate of deoxyribose degradation (Chawla et al., 2009). The scavenging activities on hydroxyl radical of the RP, MRPs and EPS were shown in Table 1. The scavenging activity increased as the concentration of MRPs increased. The scavenging activity increased from 34.7% to 76.7% as the MRPs concentration increased from 1.0 to 5.0 mg/ml. The values of *IC_50_* were 9.411 and 2.547 mg/ml for scavenging effect on OH· for the RP and MRPs, respectively. This result indicated that the MRPs have stronger scavenging activity on hydroxyl radical compared to that of RP. It revealed that the Maillard reaction products have the potential of being antioxidants in biological systems. Development of compounds capable of scavenging hydroxyl radicals to demonstrate in vitro hydroxyl radical scavenging activity of heat‐induced MRPs has been reported (Jing & Kitts, 2000). However, the value of *IC_50_* was 0.161 mg/ml for scavenging effect on OH· for the EPS. The Maillard reaction play a positive on RP only.

##### Superoxide anion scavenging activity

Numerous biological reactions generate superoxide radical (·O_2_^−^) which is a highly toxic species. Although they cannot directly initiate lipid oxidation, superoxide radical anions are potential precursors of highly reactive species, such as hydroxyl radical, and thus study of the scavenging of this radical is important (Li et al., 2008). Thus, superoxide anion scavenging activity indirectly contributes toward antioxidant potential. The scavenging activity of the RP, MRPs and EPS on superoxide radicals was shown in Table 1. At all of the concentrations tested, the scavenging activity of the RP, MRPs and EPS presented an identical increasing trend. MRPs and EPS showed strong superoxide radicals scavenging as evidenced by their low *IC_50_* value (12.488 and 0.128 mg/ml, respectively). The scavenging ability of MRPs appears to be higher than that of original RP solution. Results showed that MRPs have good antioxidant and free radicals scavenging activities and the modified protein can be a potential source of natural antioxidant. Formation of compounds that are capable of scavenging hydroxyl and superoxide anion radicals, as result of radiation induced MRPs in sugar/amino acid model systems, has been reported.

Maillard reaction products have great resistance to oxidation. The Fenton reaction system, containing ferric–ascorbate–EDTA–H_2_O_2_, generates hydroxyl radicals at a rapid rate, which in turn will react with deoxyribose and DNA, as targets of hydroxyl radical‐induced peroxidation (Jing & Kitts, 2002). The results of these investigations suggest that the EPS‐RP MRPs have the potential of exhibit varying protective effects against hydroxyl radicals in both the deoxyribose oxidation and DNA nicking tests. Similar reports are available on Maillard–based conjugation of lysozyme with polysaccharides (Scaman et al., 2006). The reaction between reducing sugars and amino acids or proteins produces strong reducing materials, such as amino reductones, which are key intermediates of the Maillard reaction (Kanokwan et al., 2006). In our study, the DG of EPS‐RP Maillard reaction is 46.8%, in the middle stage. So the reductones maybe the major production. However, this points out a direction for our future studies.

## 4 CONCLUSION

Conjugates of rice protein with EPS were prepared by hydrothermal reaction and purified by Sepharose CL‐6B column chromatography. The macromolecular components conjugated with EPSs were added by Maillard modification. The maximum fluorescence response of MRP was at 336 nm excitation wavelength and 420 nm emission wavelength, which indicated that the conjugate had fluorescence characteristics. Analysis of bands intensity in PAS reaction indicated the formation of high molecular weight conjugates by covalent binding of rice protein with EPS. The absorption peak of MRP increased at 1,180–953 cm^−1^, demonstrating the presence of carbohydrate linked to rice protein. Moreover, intensity of the bands at 3,480–3,440 cm^−1^, 1,700–1,300 cm^−1^, 1,260–1,000 cm^−1^ increased, marking the occurrence of conjugation reaction. In addition, EPS or its degradation products in the form of covalent bond cross‐linked with rice protein were further identified by Fourier transform infrared spectra. Furthermore, functional groups of –NH_2_ disappeared and those associated with MRPs including C=O, C=N and C–N increased. Due to the reducibility of EPS and the fracture of ‐S‐S‐ group of rice protein under heating and reduction conditions, the mutual conversion between disulfide bond and sulfhydryl group occurred during Maillard reaction. This also weakened the spatial structure and stability of RP. EPS or its degradation products may induce the formation of low and medium molecular weight compounds when caramelization happened at a certain pH. Thus, Maillard conjugates are a series of products with different molecular weight distribution, where melanoprotein is formed by stable covalent bonds. Due to the steric block effect of polysaccharides, Maillard aggregates dispersed after conjugating modification, and formed even tiny particles. After covalent binding of RP and EPS, the molecules spread out, reducing the molecular aggregation.

Functional properties of MRPs, including solubility, foaming activity and emulsifying ability, were greatly improved. With introducing hydrophilic groups into RP molecules, the solubility of MRPs was improved at 3–40 times at different pH. The foaming properties and foaming stability of MRP were better than those of RP, especially at pH 6.5. After conjugation modification, the original compact structure of RP was destroyed and the hidden hydrophobic groups were exposed. The conjugates were more easily dispersed on the oil–water interface, which greatly improved the emulsifying ability and emulsifying stability.

Modification of rice protein were carried out with an active polysaccharide from an endophyte by Maillard reaction. The study provided an efficient way for improving functional properties and increasing application value of plant protein.

## AUTHOR CONTRIBUTION

**Yuguang Zhao:** Conceptualization (lead); Data curation (lead); Formal analysis (lead); Resources (lead); Software (lead); Supervision (lead); Validation (lead); Visualization (lead); Writing‐original draft (lead); Writing‐review & editing (lead). **Shuhong Ye:** Funding acquisition (equal); Investigation (equal); Methodology (equal); Project administration (equal). **Huiping Wan:** Methodology (supporting). **Xingxing Zhang:** Data curation (equal); Formal analysis (equal); Resources (equal); Software (equal). **Mingqi Sun:** Data curation (supporting); Formal analysis (supporting); Software (supporting).

## ETHICAL APPROVAL

The authors declare that we have no financial and personal relationships with other people or organizations that can inappropriately influence our work, there is no professional or other personal interest of any nature or kind in any product, service and/or company that could be construed as influencing the position presented in, or the review of, the manuscript entitled. The study's protocols and procedures were ethically reviewed and approved.
